# A case of obstructive jaundice due to early carcinoma of the cystic duct protruding into the common bile duct

**DOI:** 10.1016/j.ijscr.2018.09.043

**Published:** 2018-10-04

**Authors:** Yuhki Sakuraoka, Takashi Suzuki, Genki Tanaka, Takayuki Shimizu, Takayuki Shiraki, Park Kyongha, Shozo Mori, Yukihiro Iso, Masato Kato, Taku Aoki, Keiichi Kubota, Hidetsugu Yamagishi

**Affiliations:** a Second Department of Surgery, Dokkyo Medical University, 880 Kitakobayashi, Mibu, Tochigi, 321-0293, Japan; b Department of Diagnostic Pathology, Dokkyo Medical University, 880 Kitakobayashi, Mibu, Tochigi, 321-0293, Japan

**Keywords:** US, ultrasonography, ERCP, endoscopic retrograde cholangiopancreatography, MRC, magnetic resonance cholangiography, GOT, glutamate oxaloacetate transaminase, GPT, glutamate pyruvate transaminase, γ-GTP, γ-glutamyl transpeptidase, ALP, alkaline phosphatase, CEA, carcinoembryonic antigen, CA19-9, cancer antigen 19-9, ERBD, endoscopic retrograde biliary drainage, CT, computed tomography, PET, positron emission tomography, FDG, fluorodeoxyglucose, UICC, Union for International Cancer Control, GB, gallbladder, HE, hematoxylin and eosin, pap, papillary adenocarcinoma, Case report, Early gallbladder cancer, Farrar criteria

## Abstract

**Introduction:**

Cystic duct carcinoma is a rare disease, and only 33 cases reported worldwide have completely fulfilled the criteria first established by Farrar in 1951. Here we describe an extremely rare case of early cystic duct carcinoma that fulfilled the Farrar criteria, the papillary tumour protruding into the common bile duct, leading to obstructive jaundice.

**Case presentation:**

A 76-year-old man visited a clinic with icteric conjunctivae, and was referred to our hospital for investigation of suspected obstructive jaundice. He was initially diagnosed as having a distal bile duct carcinoma on the basis of ultrasonography (US), endoscopic retrograde cholangiopancreatography (ERCP), and magnetic resonance cholangiography (MRC), and underwent pancreatoduodenectomy with regional lymphadenectomy. Macroscopic examination showed that the stalk of the papillary tumour originated from the cystic duct, and that the protruding lesion was 50 mm in size. Histopathological examination revealed the tumour to be a papillary adenocarcinoma confined within the fibromuscular layer, with no evidence of lymph node metastasis. Therefore, the final diagnosis was early cystic duct carcinoma.

**Conclusion:**

To our knowledge, this is the first case report of obstructive jaundice due to early carcinoma of the cystic duct protruding into the bile duct, with characteristics fulfilling the Farrar criteria.

## Highlights

• An extremely rare early cystic duct carcinoma.
• The protruding part was the cause of jaundice.
• There is a significant interesting macro findings in the removed sample.
• The only small part of the invasive part which reach fibro-muscular layer is revealed by the pathological findings.

## 1 Introduction

Primary carcinoma of the cystic duct is a rare disease. The cystic duct is a short and narrow tube that connects the gallbladder to the bile duct. In most cases, the origin of this malignancy is difficult to determine, which accounts for the rarity of reports. In 1951, Farrar proposed diagnostic criteria for cystic duct carcinoma. First, growth is restricted to within the cystic duct. Second, there must be no neoplastic process in the gallbladder or hepatic or common bile duct. Third, histological examination of growth must confirm the presence of carcinoma cells [1]. When we explored the previous cases by using data sources with “cystic duct carcinoma” and “Farrar criteria” as the search term beyond PubMed as well as Ichushi-Web from 1951 to 2017, only 33 cases were extracted. However, several cases of cystic duct carcinoma with invasion extending to the gallbladder neck or bile duct have been reported and classified as shown in Table 1. These new classifications considered tumour spread as well as invasion and would be more clinically useful [[2], [3], [4]] (Table1).

**Table 1:** Comparison of the four different classifications.

Classification				Age (years)	Gender (n = M/F)	Total (n)	Jaundice (n)
**Farrar (1951)**				60	21/12	33	5

**Kim et al. (2007)**				ND	ND	20	ND

**Yokoyama et al. (2008)**				ND	20/24	44	39
**Hepatic Hilum type**		**Cystic Confluence type**					
**Nakata et al. (2009)**				68	10/5	15	10
**Type I**	**Type II**	**Type III**	**Type IV**				

**Abbreviations**: ND: Data not mentioned in the report.

**Farrar criteria**: First, growth is restricted to within the cystic duct. Second, there must be no neoplastic process in the gallbladder or hepatic or common bile duct. Third, histological examination of growth must confirm the presence of carcinoma cells.

**Classification by Kim et al.**: Type I is confined to within the cystic duct. Type II means the tumor extends to the gallbladder neck or bile duct from the cystic duct side without obstructive jaundice. Type III indicates the tumor extends up to the gallbladder body or bile duct contralateral to the cystic duct opening, which then causes obstructive jaundice.

**Classification by Yokoyama et al.**: The hepatic hilum type means that the tumor mainly invades the hepatic hilum. The cystic confluence type indicates that the tumor invades the confluence of the cystic duct.

**Classification by Nakata et al.**: Type I means the tumor is located entirely within the cystic duct. Type II means that the tumor invasion has extended to the gallbladder. Type III means that the tumor invasion has extended to the common hepatic duct or common bile duct, including extension into the lumen and external invasion to the bile duct wall. Type IV means that the invasive lesion has extended to both the gallbladder and the bile duct.

We have experienced a case of early carcinoma of the cystic duct, in which invasion was limited to the fibromuscular layer and the papillary tumour protruded into the common bile duct beyond the confluence of the cystic duct to reach the common bile duct, causing obstructive jaundice. Here we describe the clinical and pathological details of this case and discuss its rarity as well as the significant discrepancies from previous classifications that it exhibited. We also review the literature and summarize the presentation and management of this rare tumour. The work has been reported in line with the SCARE criteria [5].

## 2 Case presentation

A 76-year-old man visited a local clinic with icteric conjunctivae. He had sick sinus syndrome and used a pacemaker. Blood biochemistry revealed significantly high levels of total bilirubin and transaminase, and US imaging demonstrated intrahepatic bile duct dilatation. Therefore, he was referred to our department for examination of suspected obstructive jaundice.

On admission, the patient’s body temperature was 35.9 °C, and yellowing of the conjunctivae and skin was evident. The patient had medium build, and no abnormal findings were evident in the neck or thoraco-abdominal region. Blood tests on admission showed no abnormality, but blood biochemistry revealed significant increases in the levels of transaminases and biliary enzymes (glutamate oxaloacetate transaminase (GOT): 260 U/L, glutamate pyruvate transaminase (GPT): 420 U/L, γ-glutamyl transpeptidase (γ-GTP): 1166 mU/mL, and alkaline phosphatase (ALP): 1163 U/L). The total bilirubin level was 6.0 mg/dL. Examination of tumour markers revealed a carcinoembryonic antigen (CEA) level of 3.0 ng/mL and a high level of cancer antigen 19-9 (CA19-9) (194.1 U/mL) (Table 2).

**Table 2:** Laboratory data.

Variable	Value
GOT (U/L)	260
GPT (U/L)	420
γ-GTP (mU/mL)	1166
ALP (U/L)	1163
T-Bil (mg/dL)	6.0
CEA (ng/mL)	3.0
CA19-9 (U/mL)	194.1

**Abbreviations:** GOT: glutamate oxaloacetate transaminase, GPT: glutamate pyruvate transaminase, γ-GTP: γ-glutamyl transpeptidase, ALP: alkaline phosphatase, T-Bil: total bilirubin, CEA: carcinoembryonic antigen, CA19-9: cancer antigen 19-9.

**Fig. 1:**
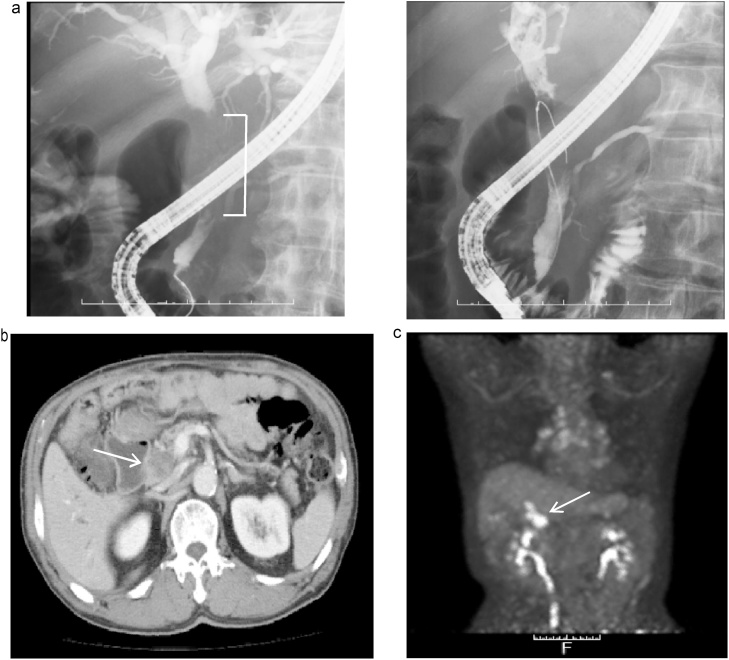
(a). Endoscopic retrograde cholangiopancreatography revealed a 5-cm-long disruption of contrast medium flow in the common bile duct, extending from the confluence of the cystic and common hepatic ducts to the distal bile duct (bracket), with significant dilation of the intrahepatic bile duct. Therefore, an endoscopic retrograde biliary drainage (ERBD) stent was inserted and placed. (b) A CT scan image revealed a contrast-enhanced protruding lesion that filled the lumen of the distal bile duct (arrow). This lesion did not extend beyond the wall of the bile duct, and neither infiltration into other organs nor clear lymphadenopathy was observed. (c) PET-CT scan revealed accumulation of FDG coinciding with the lesion in the bile duct (arrow), and no clear findings indicative of distant metastasis.

Endoscopic retrograde cholangiopancreatography (ERCP) revealed disruption of contrast medium flow from the confluence of the cystic and common hepatic ducts through the distal bile duct, as well as significant dilatation of the common and intrahepatic bile ducts. Therefore, an endoscopic retrograde biliary drainage (ERBD) stent was inserted for biliary drainage (Fig. 1a). Brush cytology at the site of distal bile duct stricture demonstrated class V (adenocarcinoma).

Abdominal computed tomography (CT) scan revealed a contrast-enhanced lesion that filled the lumen of the bile duct from inside the distal bile duct. This lesion did not extend beyond the walls of the bile duct, and neither infiltration into other organs nor no clear lymphadenopathy was observed (Fig. 1b). Positron emission tomography (PET)-CT scan revealed accumulation of fluorodeoxyglucose (FDG) that coincided with the lesion in the bile duct, and there were no clear findings of distant metastasis (Fig. 1c). Based on these results, we made a preoperative diagnosis of distal bile duct carcinoma (T1N0M0) according to the Union for International Cancer Control (UICC) classification and performed pancreatoduodenectomy with regional lymphadenectomy.

## 3 Surgical findings

The common hepatic duct was markedly dilated by a soft palpable mass approximately equivalent in size to a thumb tip. The gallbladder had swollen to fist size, and white bile was aspirated when the gallbladder was punctured. We performed pancreatoduodenectomy with lymphadenectomy, followed by reconstruction using a modification of Child’s method.

## 4 Histopathological findings

**Fig. 2:**
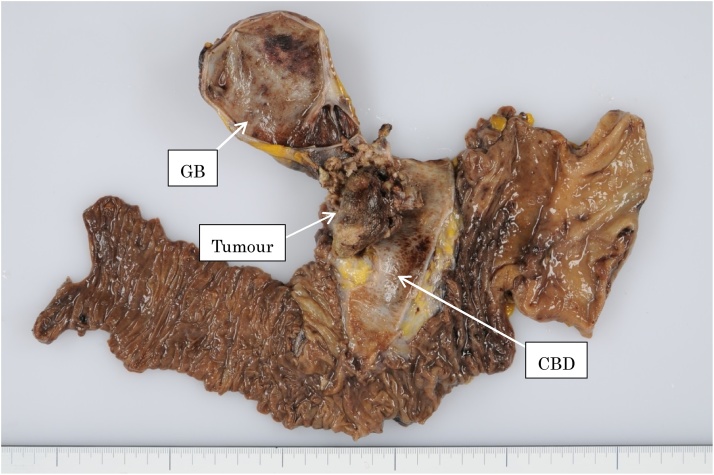
Gross features of resected specimens. In the opened bile duct, there was a papillary mass at the confluence of the cystic and common hepatic ducts. A protruding lesion (50 × 32 × 18 mm) filled the bile duct from the cystic duct through the common bile duct, and black thrombi were attached to its surface. In the lumen of the common duct, there was a large amount of blood mixed with necrotic tissue. There were no gallstones in the GB. The tumour originated at the cystic duct and was strongly connected to it by a short stem, its protruding portion reaching the bile duct easily via the confluence of the cystic duct and forming a large mass. The inflammation of the gallbladder mucosa itself was mild.

Grossly, the resected specimens revealed a papillary mass in the opened bile duct at the confluence of the cystic and common hepatic ducts. A protruding lesion (50 × 32 × 18 mm) filled the bile duct from the cystic duct through the common bile duct, and had black thrombi attached to its surface. The lumen of the common duct contained a large amount of blood mixed with necrotic material (Fig. 2). No gallstones were observed in the gallbladder (GB).

The tumour originated from the cystic duct, and bore a short, strongly connecting stem. Its protruding portion reached the bile duct through the confluence of the cystic duct and formed a large mass. The mass-forming portion had no connection with or invasion into the inner wall of the common bile duct. Moreover, class V (adenocarcinoma) was found in the fluid of the gallbladder collected during surgery. The inflammation of the gallbladder mucosa itself was mild.

**Fig. 3:**
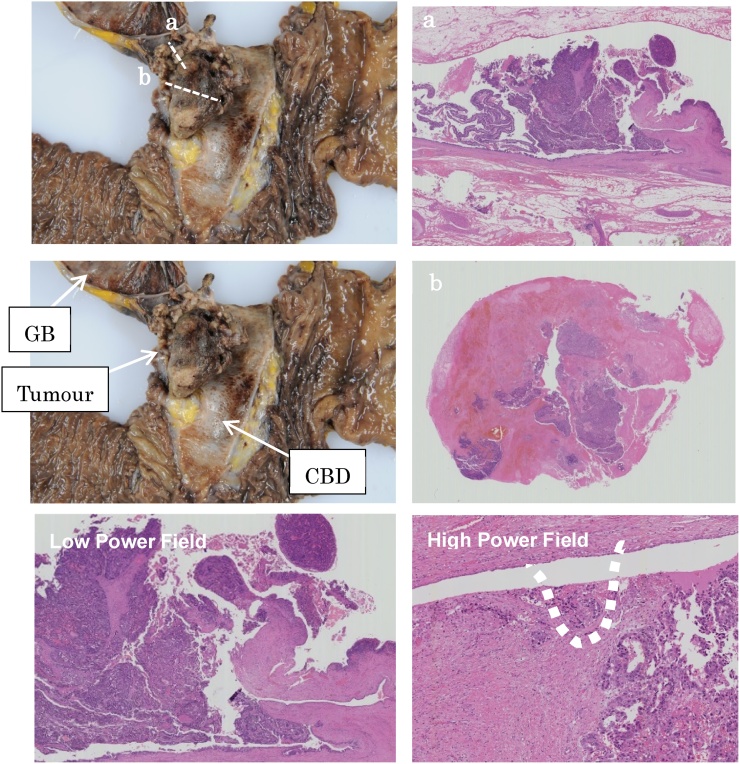
Hematoxylin and eosin staining of the tumour lesion showed adhesion of fibrin and erythrocytes around the tumour and a papillary portion within it. This tumour developed from the luminal mucosa of the cystic duct, and most of the malignant portion remained within the intraepithelial layers. The pathological diagnosis was papillary adenocarcinoma (pap), and there was no evidence of metastasis to regional lymph nodes. A comprehensive investigation revealed papillary growth of the tumour with marginal invasion to the fibro-muscular layer.

Hematoxylin and eosin (HE) staining of the tumour revealed adhesion of fibrin and erythrocytes around the tumour and papillary growth within it. The tumour had arisen from the luminal mucosa of the cystic dust, and most of the malignant portion had settled within the intraepithelial layers (Fig. 3). The pathological diagnosis was papillary adenocarcinoma (pap), without evidence of regional lymph node metastasis. A comprehensive investigation revealed papillary growth of the tumour with marginal invasion up to the fibro-muscular layer. Therefore, it was pathologically diagnosed as pT1N0M0 according to the pTNM classification [6].

## 5 Postoperative course

The patient developed a postoperative pancreatic fistula that was diagnosed as grade B based on the classification of the International Study Group on Pancreatic Fistula [7]. However, he was discharged on day 14 after surgery and is currently in good health without recurrence at 2 years and 4 months after surgery.

## 6 Discussion

The cystic duct is quite short (length around 2.5 cm) and connects the gallbladder with the common bile duct. Its walls basically consist of four layers: mucosa, a thin fibro-muscular layer, sub-serosa, and serosa. However, the wall of the cystic duct lacks proper muscle, and its structure is extremely delicate. Additionally, there is a rich network of lymphatic ducts, small veins, and nerves supplying the sub-serosa layer [8,9]. Because of their unique structure and location, carcinomas originating from the cystic duct are classified as gallbladder carcinomas and are considered to easily invade adjacent organs and lymph nodes. For this reason, most of these carcinomas are advanced when they are discovered.

Carcinomas stemming from an extrahepatic bile duct have an incidence of 0.14% among all malignancies, whereas the common bile duct is the most common site of origin (40.1%) [2]. Primary carcinoma of the cystic bile duct is extremely rare, with a reported incidence of 0.03–0.05% among all autopsy cases [10]. Smith et al. found no cases of cystic duct carcinoma among 2952 autopsy cases and 1664 biliary tract surgeries [11].

In 1951, Farrar proposed three diagnostic criteria for cystic duct carcinoma. First, growth is restricted to within the cystic duct. Second, there must be no neoplastic process in the gallbladder or the hepatic or common bile duct. Third, histological examination of tumour growth must confirm the presence of carcinoma cells. The number of primary cystic duct carcinomas that completely fulfill these criteria is extremely low because most bile duct carcinomas are tumours that show wide invasion into other organs when they are detected. So far, only 33 cases worldwide have fulfilled all of these criteria [2,[12], [13], [14], [15], [16]].

An analysis of the features of cystic duct carcinoma based on these cases showed that the disease was more common in men than in women (21:12) and that the patients ranged in age from 28 to 87 years with a mean of 60 years. With regard to symptoms, 20 (69%) of the patients had abdominal pain, and most of them had acute cholecystitis. Only 5 (14%) had jaundice, and 7 (26%) had complicating gallstone disease. In the 5 patients with jaundice, the tumour extended to the sub-serosa and lymph node metastasis was evident, leading to extrinsic compression of the bile duct and consequently obstructive jaundice. The present case fulfilled all of Farrar’s criteria. Furthermore, the papillary tumour developed within the fibro-muscular layer of the cystic duct, and the stalk protruded beyond the confluence of the cystic and common hepatic ducts, forming a large mass that reached the distal bile duct.

In relation to pathological findings, the 5 cases associated with jaundice were diagnosed as the papillary type, but none of the cases protruded into the common bile duct to occupy the lumen, as was evident in the present case. Histologically, most cases (31; 89%) were adenocarcinoma [10], but there were 2 cases of undifferentiated carcinoma [11] and one case of somatostatin-producing tumour [12]. In 13 of the cases, the degree of extension was described: the mucosa in 2, the fibro-muscular layer in 3, the sub-serosa in 2, and the serosa in 4.

In the present case, a large portion of the tumour was present within the epithelium, and some portions reached the fibro-muscular layer. In addition, the presence of fibrin and erythrocytes around the tumour proved that the tumour had occupied the lumen of the common bile duct, and that chronic inflammation and small amounts of bleeding had occurred on the surface of the papillary tumour due to abrasion caused by placement of the ERBD stent for biliary drainage (Table 3). The most important point in this case was that the malignant lesion originating from the mucosal layer infiltrated the fibro-muscular layer only at the cystic duct, and that the papillary tumour growing via a stem from the mucosa of the cystic duct reached beyond the confluence with the common bile duct without directly invading via the mucosal layer. This is why we considered this case to fulfill the Farrar criteria.

**Table 3:** Clinical features of cases fulfilling Farrar’s criteria (1941–2009).

Characteristics	Total patients (n = 33)
Age: yr	28-87(Median:60.1)
Gender: male/female	21/12
Abdominal pain	20
Jaundice	5
Stone	7

Histology	
Tubular adenocarcinoma	13
Papillary adenocarcinoma	18
Others	2

Depth	
m	2
fm	3
ss	2
se	4
Unknown Cases	22

Among the operative procedures available for cases of cystic duct carcinoma that fulfill the Farrar criteria, only cholecystectomy was performed in more than half of the cases, as preoperative diagnosis was difficult and a tumour was not suspected. We believe that for all cases where cystic duct carcinoma is suspected, at least cholecystectomy, combined resection of the bile duct, and regional lymphadenectomy should be performed. The present case was diagnosed preoperatively as distal bile duct cancer, and pancreatoduodenectomy with regional lymphadenectomy was performed. As a result, the patient obtained a favorable outcome. Among the 33 previous cases that completely fulfilled the Farrar criteria, all of the patients who underwent surgery showed favourable outcomes except for one who died within a year after surgery. Cases that fulfill all of Farrar’s criteria could possibly be considered as relatively early carcinomas of the cystic duct.

Because Farrar’s criteria are clinically limited, new classifications that are more pragmatic have been reported (Table 1). They define cystic duct carcinomas as mostly being located in the cystic duct. They also consider invasive areas so that more advanced cases can be included. In 2007, Kim et al. classified such cases into three groups based on the area of tumour infiltration and invasion: Type I is confined within the cystic duct; Type II extends to the gallbladder neck or bile duct on the cystic duct side without obstructive jaundice; and Type III extends up to the gallbladder body or bile duct on contralateral to the cystic duct opening, which then causes obstructive jaundice [3].

In 2008, Yokoyama et al. classified cases into either the hepatic hilum type or the cystic confluence type. The hepatic hilum type means that the tumour mainly invades the hepatic hilum, whereas the cystic confluence type invades the confluence of the cystic duct [4]. Nakata et al. divided cases into four groups based on the extent of spread. In Type I, the tumour is located entirely within the cystic duct, while in Type II, the tumour extends to the gallbladder. In Type III, the tumour extends to the common hepatic duct or common bile duct, including extension into the lumen and external invasion to the bile duct wall. In Type IV, the invasive lesion extends to both the gallbladder and the bile duct [2].

In spite of these clinically useful classifications, the present case could not be fully classified using Yokoyama’s criteria because the tumour did not invade either the common bile duct or the common hepatic duct. On the other hand, the classifications proposed by Kim and Nakata focused on the location of the carcinoma. From this viewpoint, our present case might be considered as Type I by both sets of criteria because adenocarcinoma was found within the cystic duct despite the protruding papillary lesion that was located mainly in the common bile duct. However, all of the Type I cases reported in the literature were unassociated with jaundice, whereas severe obstructive jaundice was evident in the present case. Thus, the present case showed considerable discrepancy from these classifications.

Obstructive jaundice was present in only 5 of the 33 cases that satisfied Farrar’s criteria. Most of these patients had lymph node metastases that produced extrinsic compression, thus hindering smooth passage of bile. Previous studies have shown that most patients with jaundice had advanced carcinomas. More specifically, Nakata et al. reported that among 15 patients with obstructive jaundice, 10 had advanced lesions [2]. These facts suggest that the present case was considerably rare because early cystic carcinoma led to obstructive jaundice attributable to the distinctive mass protruding towards the common bile duct without direct invasion through the adjacent mucosal layer.

## 7 Conclusion

We have presented a case of early carcinoma of the cystic duct in which the papillary part of the tumour protruded into the common bile duct. This is the first example of such a case to have been reported, representing an extremely rare early cystic duct carcinoma confined to the fibro-muscular layer and causing obstructive jaundice.

## Conflicts of interest

I have no financial relationships to disclose and there are not any financial and personal relationships with other people or organisations about all authors.

## Sources of funding

I have no financial relationships to disclose and there is not any sponsor with funding.

## Ethical approval

Our reported case here involves a sufficient ethnical level.

The approval has been given by the ethics committee in Dokkyo Medical University as the assigned reference number 1808-001.

## Consent

Fully informed consent was obtained with some document. Written informed consent was obtained from the patient for publication of this case report and accompanying images. A copy of the written consent is available for review by the Editor-in-Chief of this journal on request.

## Author contribution

Yuhki Sakuraoka MD, PhD: Literature review and writing the article. Keiichi Kubota MD, PhD: Editing the article and he per-formed the surgery. All authors significantly contributed to revising the manuscript. They have read and approved this manuscript.

## Registration of research studies

Our reported case here is not research study. This is a case report.

However, the approval has been given by the ethics committee in Dokkyo Medical University as the assigned reference number 1808-001

## Guarantor

Dr Yuhki Sakuraoka is the Guarantor of this report and has full responsibility to it.
